# Biosynthesis of fragrance 2-phenylethanol from sugars by *Pseudomonas* *putida*

**DOI:** 10.1186/s13068-024-02498-1

**Published:** 2024-04-02

**Authors:** Patricia Godoy, Zulema Udaondo, Estrella Duque, Juan L. Ramos

**Affiliations:** 1 Department of Environmental Protection, Estación Experimental del Zaidín, CSIC, c/ Profesor Albareda 1, 1808 Granada, Spain; 2 Department of Biomedical Informatics, University of Arkansas for Medical Science, Little Rock, AR 72205 USA

**Keywords:** 2-Phenylethanol, l-Phenylalanine, *Pseudomonasputida*, Microbial production, Biosynthetic pathways, Chemical mutagenesis, 2G sugars

## Abstract

**Background:**

Petrochemicals contribute to environmental issues, with concerns ranging from energy consumption and carbon emission to pollution. In contrast, microbial biorefineries offer eco-friendly alternatives. The solvent-tolerant *Pseudomonas* *putida* DOT-T1E serves as a suitable host for producing aromatic compounds, specifically l-phenylalanine and its derivative, 2-phenylethanol (2-PE), which find widespread applications in various industries.

**Results:**

This study focuses on enhancing 2-PE production in two l-phenylalanine overproducing strains of DOT-T1E, namely CM12-5 and CM12-5Δ*gcd* (*xylABE*), which grow with glucose and glucose-xylose, respectively. To synthesize 2-PE from l-phenylalanine, these strains were transformed with plasmid pPE-1, bearing the Ehrlich pathway genes, and it was found higher 2-PE production with glucose (about 50–60 ppm) than with xylose (< 3 ppm). To understand the limiting factors, we tested the addition of phenylalanine and intermediates from the Ehrlich and shikimate pathways. The results identified intracellular l-phenylalanine as a key limiting factor for 2-PE production. To overcame this limitation, a chorismate mutase/prephenate dehydratase variant—insentive to feedback inhibition by aromatic amino acids—was introduced in the producing strains. This led to increased l-phenylalanine production and subsequently produced more 2-PE (100 ppm). Random mutagenesis of the strains also produced strains with higher l-phenylalanine titers and increased 2-PE production (up to 120 ppm). The improvements resulted from preventing dead-end product accumulation from shikimate and limiting the catabolism of potential pathway intermediates in the Ehrlich pathway. The study explored agricultural waste substrates, such as corn stover, sugarcane straw and corn-syrup as potential C sources. The best results were obtained using 2G substrates at 3% (between 82 and 100 ppm 2-PE), with glucose being the preferred sugar for 2-PE production among the monomeric sugars in these substrates.

**Conclusions:**

The findings of this study offer strategies to enhance phenylalanine production, a key substrate for the synthesis of aromatic compounds. The ability of *P.* *putida* DOT-T1E to thrive with various C-sources and its tolerance to substrates, products, and potential toxicants in industrial wastes, are highlighted. The study identified and overcome possible bottlenecks for 2-PE production. Ultimately, the strains have potential to become efficient microbial platforms for synthesizing 2-PE from agro-industrial waste materials.

**Supplementary Information:**

The online version contains supplementary material available at 10.1186/s13068-024-02498-1.

## Introduction

Petrochemicals are one of the largest groups of compounds used in the manufacture of thousands of daily used goods and are one of the largest contributors to energy consumption and carbon dioxide emissions. In fact, it has been estimated that the petrochemical industry consumes about 14% of the oil and gas used in the world. This is because the production of petrochemicals requires extremely high pressures and temperatures (400–900 °C) [1]. Concomitantly, the petrochemical industry produces a wide range of waste products that pollute the air, water and soil.

One promising way to reduce the negative impact of the chemical industry is the development of microbial platforms that, through fermentative processes, can produce added-value chemicals under room temperature and ambient pressure conditions, and are environmentally friendly [2–5]. However, a number of industrially relevant chemicals are toxic for many but not all microorganisms, and adequate production platforms need to be in place [2–6]. Therefore, choosing the right chassis for the implementation of synthetic routes is of utmost importance for the success of the industry, as productivity is key to the economics of the project [7].

In the case of the bio-production of aromatic compounds, such as l-phenylalanine, l-tyrosine and their derivatives, a useful platform is *Pseudomonas* *putida,* because certain strains of this species are naturally equipped with numerous traits that allow them to thrive in the presence of high concentrations of a wide range of aromatic compounds [8, 9]. l-Phenylalanine is a relevant intermediate for the synthesis of aromatic compounds of industrial value such as styrene, cinnamic acid, and aromatic alcohols, such as 2-phenylethanol (2-PE). 2-PE is a valuable ingredient in the cosmetic and perfume industries [10] and it is also used as the substrate for the synthesis of additives and preservatives in the food industry and the pharmaceutical sector [11, 12]. Although 2-PE can be extracted from certain flowers, the majority of 2-PE is chemically synthesized [13, 14] in processes that use toxic solvents and catalysts, aggressive reaction conditions, which yield high amounts of undesirable side-products which decrease the quality of the resulting 2-PE.

**Fig. 1:**
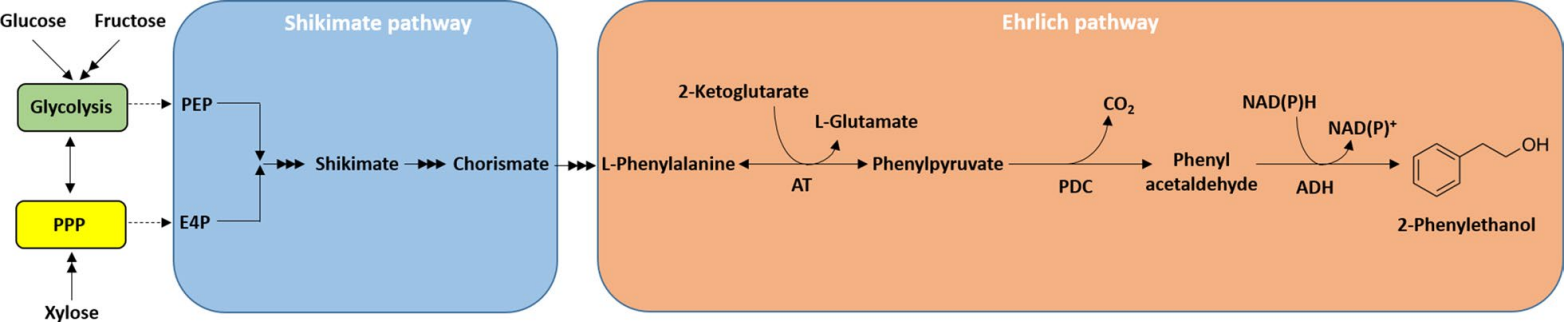
2-Phenylethanol biosynthesis from glucose through the Ehrlich pathway. Glucose metabolized through the Etner-Doudoroff and pentose phosphate pathway (PPP) yield; phosphoenolpyruvate (PEP) and erythrose-4-phosphate E4P; which are channeled through the shikimate/chorismate to phenylalanine (PE). Then, the Ehrlich pathway enzyme produces 2-PE. AT, transaminase; PDC, phenylpyruvate decarboxylase; ADH, alcohol dehydrogenase. (Adapted from Qian et al. [20])

On the contrary, the biological production of 2-PE is an alternative eco-friendly process that can be achieved through different pathways, including the phenylacetaldehyde synthase pathway, the phenylethylamine pathway, and the Ehrlich pathway, through which 2-PE is naturally produced by different yeasts, such as *Saccharomyces* *cerevisiae*, *Kluyveromyces* *marxianus,* *Pichia* *fermentas* and *Yarrowia* *lipolitica* [15–22]. De novo production of 2-PE from glucose by engineered bacteria has been achieved with *Escherichia* *coli,* *Bacillus* *licheniformis,* *Enterobacter* sp. and *Pseudomonas* *putida* [20, 23–26]; however, the titers are often low and industrial sugar sources have not been used. In the Ehrlich pathway (Fig. 1), stoichiometric conversion of l-phenylalanine into 2-PE takes place in three steps [27]: Firstly, deamination of l-phenylalanine to phenylpyruvate by a transaminase; secondly, decarboxylation of phenylpyruvate to phenylacetaldehyde by a phenylpyruvate decarboxylase; and thirdly, reduction of phenylacetaldehyde to 2-PE by an alcohol dehydrogenase (Fig. 1).

*Pseudomonas* *putida* CM12-5,—a solvent-tolerant l-phenylalanine producer—is a *P.* *putida* DOT-T1E derivative generated by using a combination of genetic strategies, namely, (1) chemical mutagenesis and the selection of clones resistant to toxic analogues of l-phenylalanine and (2) site-directed insertional inactivation of genes involved in l-phenylalanine catabolism [24]. When *P.* *putida* DOT-T1E CM12-5 was transformed with the plasmid pPE-1, bearing a histidinol phosphate transferase (PP_0967 from *P.* *putida* KT2440), a phenylpyruvate decarboxylase (*kdc* from *Rhodospirillum* *rubrum*) and a native alcohol dehydrogenase (T1E_5478, *adh* from *P.* *putida* DOT-T1E), the resulting strain was able to produce 2-PE from glucose [24]. In addition to glucose, *Pseudomonas* *putida* also uses fructose as the sole C-source [28, 29], another major sugar used in industry; however, it cannot use xylose, the second most abundant monomeric sugar after glucose in enzymatic hydrolysates of lignocellulosic residues (2G sugars) [30]. Several research groups have constructed variants of *P.* *putida* that use xylose as the sole C-source, this requires the inactivation of the *gcd* gene encoding a glucose/xylose dehydrogenase that convert xylose in dead-end xylonate and incorporation of xylose transport (*xylE*) and catabolic genes (*xylAB*) that convert xylose into xylose-5-phosphate that enters into the pentose phosphate cycle [31–33].

The aims of this study were: (i) to study the synthesis of 2-phenylethanol by solvent-tolerant *Pseudomonas* *putida* DOT-T1E derivatives with various carbon sources; (ii) to identify the limiting step in the biosynthesis of phenylalanine and its conversion to 2-PE in this *P.* *putida* chassis; (iii) to generate mutant derivatives that are more efficient in the biosynthesis of 2-PE, and (iv) to test different industrial carbon stocks for the synthesis of 2-PE. This study identifies the synthesis of l-phenylalanine as the main bottleneck in the production of 2-PE and that prevention of the production of dead-end products from shikimate increased the intracellular levels of l-phenylalanine and its subsequent conversion to 2-PE. Glucose has been identified as the preferred sugar in industrial sources for the biosynthesis of the aromatic alcohol.

## Results

### Synthesis of 2-PE from glucose and xylose by *P. putida* CM12-5 derivatives

**Fig. 2:**
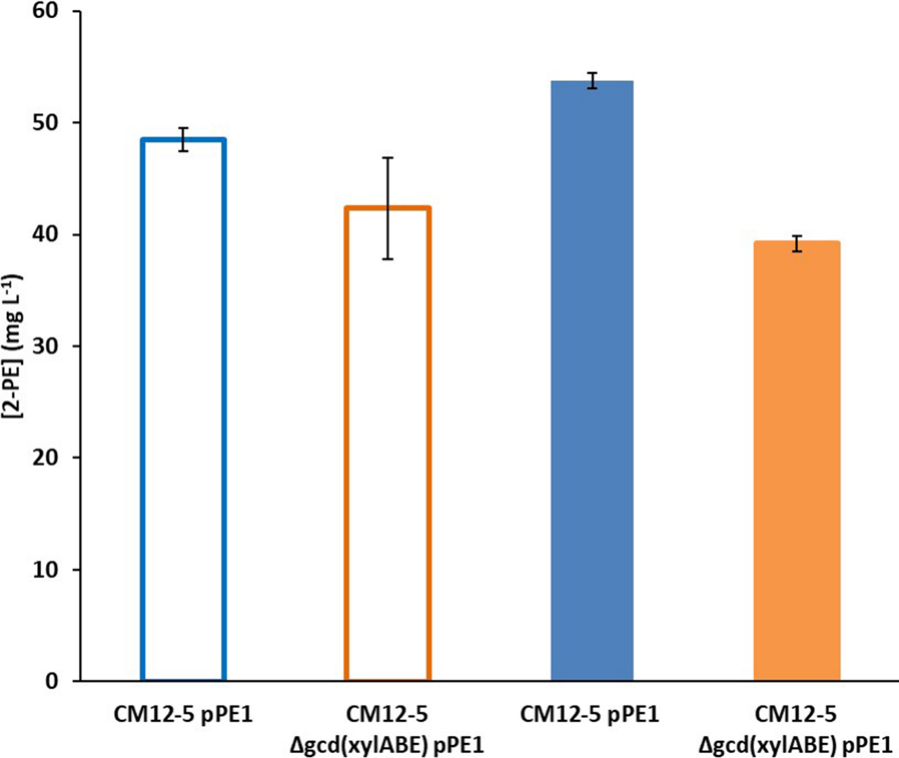
Production of 2-PE in the presence of glucose (open bars) or mixture of glucose:xylose (closed bars) as the C source. *P.* *putida* CM12-5 (pPE-1) and CM12-5Δ*gcd* (*xylABE*) (pPE-1) cells were grown overnight with glucose as the C source. The cultures were then washed and diluted to DO_660_ of 0.1 diluted in fresh medium with glucose (0.5% w/v) or glucose:xylose (0.375% w/v:0.125% w/v) as the C source, and 2-PE production was measured after 24 h incubation. The figure shows the average of three independent assays

The two L-phenylalanine producing strains CM12-5 and CM12-5Δ*gcd* (*xylABE*) that metabolize glucose and glucose and xylose, respectively, were transformed or not with plasmid pPE-1 and synthesis of 2-PE by the four strains tested with glucose, xylose and mixtures of glucose:xylose (3:1) in assays that lasted 24 h. Our results are shown in Fig. 2. The two strains without the pPE-1 plasmid did not produce 2-PE as expected (not shown). We found that the *P.* *putida* CM12-5 (pPE-1) and *P.* *putida* CM12-5Δ*gcd* (*xylABE*) (pPE-1) produced about 50 ppm 2-PE when glucose was the growth substrate (Fig. 2). However, with xylose, negligible 2-PE accumulated, i.e., less than 3 ppm was detected in the culture medium (not shown). When glucose and xylose were present simultaneously, a production level of 2-PE similar to that obtained with glucose alone was achieved (Fig. 2). These strains accumulated low amounts of phenylalanine in the culture medium (< 3 ppm) under all growth conditions (not shown), which suggests that almost all phenylalanine produced intracellularly was channeled towards 2-PE production.

Since xylose is metabolized through the pentose phosphate pathway (PPP), it is likely that phosphoenolpyruvate (PEP) levels in CM12-5Δ*gcd* (*xylABE*) are limiting the operation of the shikimate pathway (Fig. 1), which is critical for production of l-phenylalanine and its subsequent channeling towards 2-PE.

### Effect of the addition of the Ehrlich pathway and shikimate pathway intermediates on 2-PE production

**Fig. 3:**
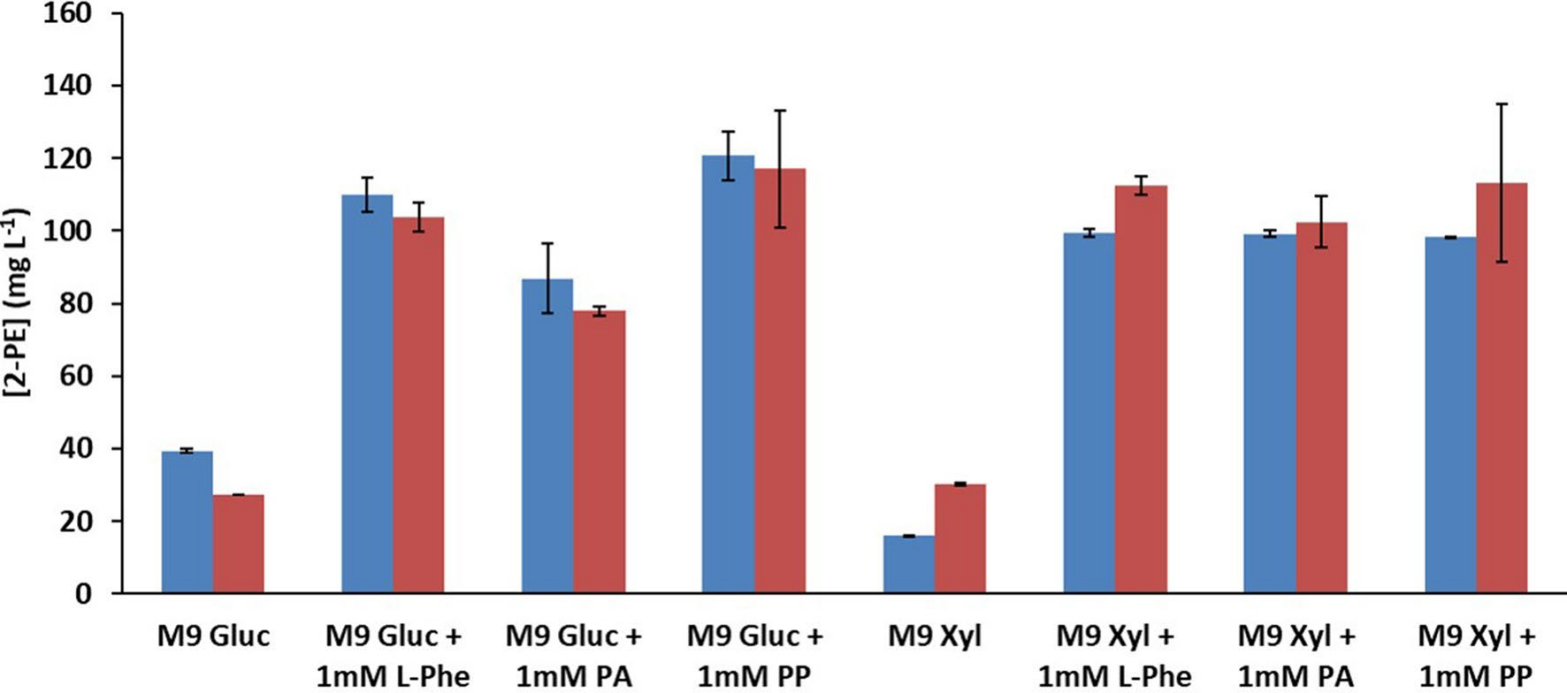
Production of 2-PE by resting cells in the presence of Ehrlich pathway intermediates: l-phenylalanine (l-Phe), phenylacetaldehyde (PA) or phenylpyruvate (PP). CM12-5 (pPE-1) (blue bars) and CM12-5Δ*gcd* (*xylABE*) (pPE-1) (red bars) cells were grown in the presence of glucose as the C source until reaching a DO_660_ of 1, the cultures were then washed and concentrated in 1xM9 + 0.5% (w/v) glucose or xylose to reach a DO_660_ of about 10, and the effect of 1 mM l-Phe PA or PP supplements on 2-PE production was measured after 24 h of incubation. The figure shows the average values of three independent assays

To determine if the limiting factor in the process was related to the production of the primary substrate, l-phenylalanine (l-Phe), or pathway intermediates, i.e., phenyl acetaldehyde (PA) and phenylpyruvate (PP), we carried out resting cells assays in which 1 mM l-Phe (165 ppm), 1 mM PA (120 ppm) or 1 mM PP (186 ppm) were added to cultures of CM12-5 (pPE-1) and CM12-5Δ*gcd* (*xylABE*) (pPE-1), in a medium with glucose or with xylose as the C source. Figure 3 shows that, regardless of the C source used (glucose or xylose), 2-PE production reached concentrations of about 100–120 ppm 2-PE in both strains, increases that represent production about 200% higher than those obtained in the absence of Ehrlich pathway intermediates. Therefore, the transformation of l-Phe into 2-PE through the Ehrlich pathway was not compromised. This suggests that the initial amount of l-Phe in the cells is the main limiting factor in the production of 2-PE.

On the other hand, l-phenylalanine in *P.* *putida* is made through the shikimate pathway, in which shikimate and chorismate are intermediates (Fig. 1), and hence we tested whether the addition of 1 mM shikimate (174 ppm) or 1 mM chorismate (226 ppm) would also lead to an increase in 2-PE production. Supplementation with chorismate to CM12-5 (pPE-1) and CM12-5Δ*gcd* (*xylABE*) (pPE-1) growing with glucose or xylose (Table 1) led to production levels of about 1 ppm 105 ppm 2-PE (Table 1), a value similar to that reached when Ehrlich pathway intermediates were added. However, this was not the case with shikimate, as it was only partially consumed by the cells, and also because it was biotransformed in side dead-end products (3-hydroxyshikimic acid, quinate and acetate) both with glucose or xylose as the C source (data not shown). This implies that an appropriate channeling of shikimate to chorismate is a critical issue in the synthesis of 2-PE by this host platform. Hence, our data support the hypothesis that the intracellular levels of l-Phe maybe the limiting step in the biosynthesis of 2-PE. Moreover, this was particularly noticeable, when xylose was used as the sole C source, since with this C-source a clear limitation of the available intermediates for the synthesis of l-Phe was identified.

**Table 1:** Effect of shikimate pathway intermediates chorismic acid (CA) and shikimic acid (SA) on the synthesis of 2-PE in *P.* *putida* CM12-5 (pPE-1) and CM12-5Δ*gcd* (*xylABE*) (pPE-1) strains

C source	Supplement	[2-PE] (mg L^−1^)	
		CM12-5 (pPE-1)	CM12-5Δ*gcd* (*xylABE*) (pPE-1)
M9 Glucose	None	39.3 ± 0.5	27.3 ± 0.1
	+ 1 mM CA	104.3 ± 1.8	120.5 ± 0.9
	+ 1 mM SA	72.9 ± 3.8	83.5 ± 2.2
M9 Xylose	None	16.0 ± 0.3	30.1 ± 0.4
	+ 1 mM CA	105.4 ± 1.7	124.8 ± 1.6
	+ 1 mM SA	51.4 ± 1.0	88.6 ± 3.7

The assay was carried as described in the legend for Fig. 3 except that 1 mM chorismic acid (CA) or 1 mM shikimic acid (SA) were added. Values and standard deviation are the average of three independent assays

### Increasing phenylalanine production and 2-PE levels in *P. putida*

**Fig. 4:**
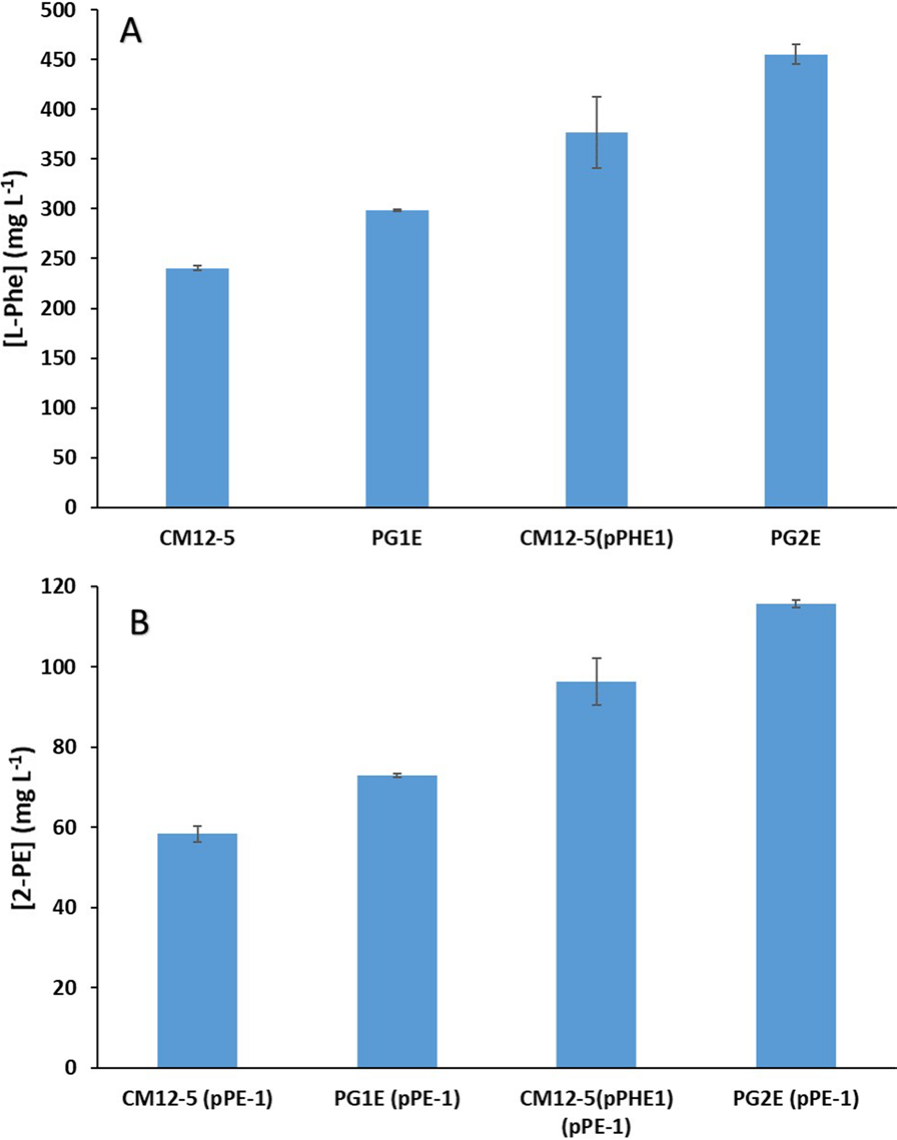
l-Phe production and 2-PE production from 1.5% (w/v) glucose of *Pseudomonas* *putida* CM12-5 derivative strains without and with the pPE-1 plasmid. Cells were grown overnight with glucose as the C source, then the cultures were diluted to DO_660_ of 0.1 in fresh medium with 1.5% (w/v) glucose, and l-Phe production (panel **A**) or 2-PE (panel **B**) was measured after 24 h incubation. The figure shows the average values of three independent assays

It has been described that l-Phe exerts feedback inhibition of its own synthesis through interaction of the amino acid with the R-domain of chorismate mutase/prephenate dehydratase, PheA, a bifunctional enzyme in the shikimate pathway for the synthesis of aromatic amino acids [34]. Molina-Santiago et al. [24] constructed PheA^fbr^, a PheA mutant variant in which the R-domain of PheA was deleted and the allosteric sensitivity to phenylalanine was eliminated. This PheA^fbr^ mutant variant was expressed in plasmid pPHE1. This study confirmed that CM12-5 (pPHE1) accumulated higher titers of L-phenylalanine in the culture medium i.e., up to 350 ± 10 mg L^−1^ versus around 300 ± 5 mg L^−1^ for CM12-5 (Fig. 4A). Then, CM12-5 (pPHE1) was transformed with pPE-1 and 2-PE measured in the medium. It was found that 2-PE increased from about 50–60 ppm to near 95 ± 5 ppm (Fig. 4B), production levels similar to those achieved with exogenous supplementation of l-Phe in resting cell assays. This confirmed that the limitation in the production of l-Phe is an obvious step in the production of 2-PE.

Thereafter, it was decided to search for clones that can produce more l-Phe than the CM12-5 and CM12-5 (pPHE1) strains. To this end, the *P.* *putida* CM12-5 and CM12-5 (pPHE1) strains were subjected to random mutagenesis with EMS and selection of clones resistant to high concentrations (> 3 mg mL^−1^) of the l-phenylalanine analogue *p*-fluoro-phenylalanine (FPA), the mutagenesis was carried out as described in Materials and Methods. One clone of each strain (named PG1E and PG2E), which were able to accumulate about 300 and 450 ppm L-Phe, respectively, when grown with 1.5% (w/v) glucose (Fig. 4A) were kept. These overproducer strains were subsequently transformed with pPE-1 plasmid and 2-PE accumulation determined. With *P.* *putida* PG1E, around 100 ppm 2-PE accumulated and the highest levels of 2-PE were reached with PG2E that accumulated at close to 120 ppm (Fig. 4B).

As the highest l-Phe and 2-PE productions were achieved with PG2E strain, it was decided to sequence its genome to determine which genomic changes were responsible for l-Phe overproduction. When comparing the genome of this l-Phe overproducer derivative PG2E with DOT-T1E genome, we found that, apart from the five mutations involved in l-Phe degradation pathways generated by direct mutagenesis and described in Molina-Santiago et al. [24], up to 400 SNP (see Additional file 2: Table S1) were found. To refine the study, the SNP in the genes that encode proteins related to l-Phe metabolism (Additional file 4: Table S3) were analyzed. A H431P change in shikimate dehydrogenase (QuiA) that may prevent the misrouting of shikimate to 3-hydroxyskimate was identified; together with a set of mutations in genes encoding enzymes of the phenylacetic acid (PAA) degradation pathway. As suggested by L324P in PaaH_2, Y154C in PaaK, V154A and F284L in PaaA (Additional file 3: Table S2). Indeed, due to the mutations in the *paa* genes, the PG2E strain lost the ability to metabolize PAA (see Additional file 1: Fig. S1). Therefore, the higher accumulation of l-Phe in the strain producing the highest l-Phe level, and the subsequent 2-PE obtained, may be related to a more efficient channeling of shikimate to l-Phe.

### The use of agricultural waste products as feedstocks for 2-PE production

To explore the potential of *Pseudomonas* *putida* as a chassis to produce 2-PE from 2G substrates, the capability of producing 2-PE from corn stover (PCS) and sugar cane straw (PSCS) hydrolysates, prepared as described in Materials and Methods was assayed. To this end, *P.* *putida* CM12-5 (pPE-1), CM12-5(pPHE1) (pPE-1), CM12-5(pPHE1)-FPA (pPE-1) and CM12-5Δ*gcd* (*xylABE*) (pPE-1) strains were grown in M8 minimal medium with KNO_3_ as the N source, and 3% (w/v) PCS or PSCS. 2-PE production levels were determined after 24 h and the results are shown in Table 2. All the strains produced between 82 and 110 mg L^−1^ 2-PE, either from 3% PCS or 3% PSCS hydrolysates. The concentration of sugars was also considered in molar terms to estimate molar yields, which were in the range of 8.6–11.1% regarding glucose utilization (Table 2).

**Table 2:** 2-PE production from 2G lignocellulosic hydrolysates PCS and PSCS in *Pseudomonas* *putida* CM12-5 derivative strains

		3% PCS				3% PSCS			
		(pPE-1)	(pPHE1) (pPE-1)	PG2E (pPE-1)	Δ*gcd*(*xylABE*) (pPE-1)	(pPE-1)	(pPHE1) (pPE-1)	PG2E (pPE-1)	Δ*gcd*(*xylABE*) (pPE-1)
mg L^−1^	Initial glucose	1630 ± 78	1753 ± 130	1638 ± 107	1562 ± 87	1533 ± 111	1576 ± 99	1576 ± 166	1576 ± 135
	Consumed Glucose	1334 ± 32	1682 ± 149	1456 ± 131	1425 ± 116	1441 ± 121	1548 ± 103	1358 ± 247	1443 ± 195
	Produced 2-PE	86 ± 19	109 ± 17	110 ± 25	85 ± 14	82 ± 15	97 ± 16	93 ± 15	84 ± 11
	Initial xylose	2596 ± 178	2627 ± 59	2600 ± 37	2633 ± 115	2034 ± 203	2305 ± 18	1910 ± 394	2065 ± 205
	Consumed xylose	1990 ± 232	1690 ± 230	1302 ± 431	1978 ± 254	1698 ± 380	1788 ± 102	1367 ± 305	1574 ± 181
mM	Initial glucose	9.05 ± 0.43	9.20 ± 0.72	9.09 ± 0.59	8.67 ± 0.48	8.51 ± 0.61	8.75 ± 0.55	8.75 ± 0.92	8.75 ± 0.75
	Consumed Glucose	7.40 ± 0.18	8.68 ± 0.83	8.08 ± 0.73	7.91 ± 0.64	8.00 ± 0.67	8.59 ± 0.57	7.54 ± 1.37	8.01 ± 1.08
	Produced 2-PE	0.70 ± 0.15	0.82 ± 0.14	0.90 ± 0.21	0.69 ± 0.12	0.67 ± 0.12	0.80 ± 0.13	0.77 ± 0.12	0.69 ± 0.09
	Initial xylose	17.29 ± 1.19	17.50 ± 0.40	17.32 ± 0.25	17.54 ± 0.77	13.55 ± 1.35	15.36 ± 0.12	12.73 ± 2.62	13.76 ± 1.36
	Consumed xylose	13.26 ± 1.55	11.26 ± 1.53	8.67 ± 2.87	13.18 ± 1.69	11.31 ± 2.53	11.91 ± 0.68	9.11 ± 2.03	10.49 ± 1.21
(G) Molar yield (%)		8.85 ± 1.68	9.51 ± 1.82	11.14 ± 1.76	8.80 ± 1.66	8.68 ± 1.28	9.24 ± 0.89	10.17 ± 0.26	8.64 ± 1.06

The assays were carried out as described in M8 minimal medium with 10 mM KNO_3_ and 3% (w/v) of hydrolysated PCS or PSCS. Glucose and xylose were determined at the beginning of the assay and 24 h later. The concentration of 2-PE was determined at the end of the assay. The values and standard deviation are the average of three independent assays

Similar assays were also done with all of these 2-PE producing strains in M9 minimal medium, but using 1G substrates as the C source, such as corn syrup, which was used at 1/80 (v/v). Our results are shown in Table 3. Surprisingly, the total production of 2-PE with 1G substrates was lower than with 2G substrates, since these strains accumulated between 10 and 84 mg L^−1^ of 2-PE and the molar yields were below 4.8%. This is in accordance with low l-Phe accumulation in the culture medium when the CM12-5 strain was grown with fructose instead of glucose as the sole C source (not shown). This may be due to the high phosphoenolpyruvate (PEP) and energy requirements for the fructose intake through the PTS active transport system [35, 36]. The lower yield agrees with the fact that glucose and fructose were not consumed completely after 48 h; in fact, on average, only 30% of glucose was depleted, while 5% of the fructose was consumed (not shown).

**Table 3:** 2-PE production from corn syrup (CS) in *Pseudomonas* *putida* CM12-5 derivative strains

		CS 1/80 (v/v)			
		(pPE-1)	(pPHE1) (pPE-1)	PG2E (pPE-1)	Δ*gcd* (*xylABE*) (pPE-1)
mg L^−1^	Initial glucose	4725 ± 94	4737 ± 95	4722 ± 89	4729 ± 100
	Consumed glucose	2375 ± 48	2117 ± 42	2579 ± 52	2204 ± 44
	Initial fructose	6351 ± 127	6323 ± 162	6318 ± 120	6349 ± 127
	Consumed fructose	429 ± 9	357 ± 7	273 ± 5	474 ± 9
	Produced 2-PE	28.7 ± 0.57	43.8 ± 0.87	84.1 ± 1.68	10.5 ± 0.21
mM	Initial glucose	26 ± 0.52	26 ± 0.53	26 ± 0.52	26 ± 0.52
	Consumed glucose	13 ± 0.26	12 ± 0.24	14 ± 0.28	12 ± 0.24
	Initial fructose	35.3 ± 0.71	35.3 ± 0.70	35.3 ± 0.70	35.3 ± 0.71
	Consumed fructose	2.4 ± 0.05	2.0 ± 0.04	1.5 ± 0.03	2.6 ± 0.05
	Produced 2-PE	0.23 ± 0.01	0.36 ± 0.02	0.69 ± 0.03	0.09 ± 0.01
(G) Molar yield (%)		1.78 ± 0.03	3.05 ± 0.05	4.81 ± 0.09	0.70 ± 0.01

The assays were carried as described in the footnote for Table 3 except that corn syrup was used. Glucose and fructose were determined at t = 0 and 24 h, and 2-PE was measured at the end of the assays. The values and standard deviation are the average of three independent assays

Therefore, industrial substrates such as corn syrup (1G substrate), and corn stover and sugar-cane straw hydrolysates (2G substrates) are suitable for using in 2-PE production in a more sustainable and environmentally friendly way; however, amongst its components, glucose is preferably consumed over fructose or xylose, and the presence fructose may slow this process down.

## Discussion

The interest in the development of technology for green chemistry increased after a series of studies revealed that nearly two thirds of all chemical products in use could be made through the fermentation of sugars obtained from seeds (i.e., corn, wheat, and others), plant residues, organic waste and lignin [37–40]. The OECD and other agencies are aiming to transition 30% of the total petrochemical industry to renewable sources by 2050 [41–44].

Several research groups have put great effort into developing *Pseudomonas* *putida* as a chassis for the biochemical production of aromatic compounds because of its metabolic properties, and its ability to tolerate stress and high concentrations of solvents [24, 33, 45–49]. Our specific overall aim is the construction of solvent-tolerant *Pseudomonas* strains that can perform optimally and achieve high yields of industrially valuable chemicals, such as 2-PE, *trans*-cinnamic acid and styrene. We have estimated that these cell factories can reduce CO_2_ emissions by about 90% compared to current petrochemical-based methods.

In this study, the molecular basis for 2-PE production when *Pseudomona*s is used as a chassis were analyzed. Production of 2-PE can be considered to take place in two sets of reactions: one that leads to synthesis of the primary substrate, L-Phe, and a second set that uses the Ehrlich pathway to produce 2-PE. It was found that, whether intracellularly made or exogenously supplied, L-Phe is stoichiometrically converted on 2-PE. The genes of the Ehrlich pathway were expressed in pPE-1 plasmid from the regulated Pm promoter [50, 51], whose expression is driven by XylS in the presence of alkylbenzoates in plasmid pPE-1, or from a constitutively expressed *lac* promoter in plasmid pPE-2 (not shown). Our unpublished results revealed that the regulated expression of the Ehrlich pathway yielded more consistent production than the constitutive expression, probably due to the generation of instability, as production decreased with time (P. Godoy, unpublished results).

The limiting factor in the 2-PE production process was linked to the production of the primary substrate, phenylalanine (L-Phe). The limited flux toward the shikimate synthesis pathway seems to be the main bottleneck for the production of aromatic chemicals, because it is highly regulated at transcriptional and enzymatic levels [25, 52]. A key feature in a wide range of l-phenylalanine producers is the need to overcome the feedback inhibition by l-Phe of the bifunctional chorismate mutase/prephenate dehydratase (PheA) enzyme [24, 25]. This fact has been confirmed in this study, as the expression of the PheA^fbr^ variant lacking the R-domain expressed in plasmid pPHE1 [24] led to significant increases in L-phenylalanine accumulation in the medium. Another step is to increase the availability of shikimate precursors phosphoenolpyruvate (PEP) and erythrose-4-phosphate (E4P) [25, 53–57]. This study shows that xylose is a less efficient C-source for l-Phe production than glucose, which is probably related to the low availability of PEP in cells growing on xylose, which limits the C flow towards the shikimate pathway. In addition, while the Ehrlich pathway intermediates are stoichiometrically transformed into 2-PE, we observed that part of shikimate made in the cells may divert to dead-end 3-hydroxyshikimate. This was indeed confirmed when clones able to produce higher amounts of L-Phe were isolated upon a round of random chemical mutagenesis that resulted in the identification of a mutation in shikimate dehydrogenase that prevents 3-hydroxyshikimate accumulation, and limited metabolism through the phenylacetic acid degradation pathway.

Lignocellulose from agricultural residues or from urban organic waste are expected to represent a relevant raw material for the production of chemicals. Thus, *P.* *putida* DOT-T1E and the l-Phe overproducing strains were engineered to bear the machinery involved in xylose uptake and catabolism and also for the 2-PE production genes. However, low production of l-Phe and 2-PE from xylose was found, in spite xylose being metabolized through the pentose phosphate pathway that generates E4P, which is a precursor of the shikimate pathway (see Fig. 1). Due to the fact that when l-Phe or Ehrlich pathway intermediates were added to cells grown of xylose, they were stoichiometrically converted to 2-PE by CM12-5 derivatives; channeling xylose to shikimate seems to be a key limiting step in 2G conversion of C5 sugars into 2-PE. Furthermore, PEP is also limiting due to its channeling to pyruvate that is eventually transformed into acetyl-CoA and enters the TCA cycle [25, 56, 57]. Taking all these facts into consideration, it is clear that it is necessary to re-organize the PEP-4EP metabolic processes when xylose is used as the C-source.

It is known that 2G hydrolysates, in addition to sugars, contain a wide range of toxic chemicals such as furfural, 5-hydroxymethylfurfural and others ([30] and Additional file 4: Table S3). These toxic compounds are originated from partial degradation of sugars during steam-explosion in the intensive physicochemical pre-treatment of lignocellulosic material [30]. These compounds affect the viability of some microorganisms used for biotransformation of 2G substrates [20, 58, 59]. In this context, *P.* *putida* DOT-T1E has been shown to grow in the presence of > 50, 25 and 25 mM of furfural, 3-hydroxymethylfurfural and syringic acid, respectively, in agreement with the high tolerance of this strain to toxic compounds, although their combination enhanced toxicity. DOT-T1E and its derivatives thrive at maximal rate with up to 3% (w/v) of lignocellulose substrates, and adaptative evolution assays (ALE) in our lab ended with evolved clones adapted to tolerate up to 11% (w/v) lignocellulosic substrates (Juan L. Ramos, unpublished results). We are focusing our efforts on enhancing the intracellular production of l-Phe as a primary substrate for the synthesis of aromatic compounds and the evolution of the strain to thrive with a wide range of C-sources and tolerance to substrates, products and possible toxicants in industrial wastes to further identify and engineer possible bottlenecks for 2-PE production. We strongly support the idea that *P.* *putida* DOT-T1E could become an efficient microbial cell factory for the production of aromatic compounds from agro-industrial waste material.

## Conclusions

Petrochemicals exacerbate environmental issues by necessitating substantial energy inputs for their production, contributing to carbon emissions, and directly or indirectly causing pollution. In contrast, we have identified *P.* *putida* DOT-T1E as a promising biorefinery for the sustainable and environmentally friendly production of aromatic compounds, specifically 2-PE, from various sugars. This approach stands in contrast to conventional chemical methods.

The strains CM12-5 and CM12-5Δ*gcd,* designed to overproduce l-phenylalanine, demonstrated the capability to convert this amino acid into 2-PE upon the expression of Ehrlich pathway genes from plasmid pPE-1. The intracellular concentration of l-phenylalanine was identified as a critical limiting factor in 2-PE production. The introduction of a mutant variant of chorismate mutase/prephenate dehydratase, bypassed feedback inhibition by aromatic amino acids, and significantly enhanced l-phenylalanine production and, consequently, boosted 2-PE yields. Furthermore, the application of random mutagenesis led to strains exhibiting elevated l-phenylalanine production, resulting in increased 2-PE production.

In the realm of industrial applications, we explored the use of agricultural waste substrates such as corn stover, sugar cane straw, and corn syrup as potential carbon sources. Regardless of the waste source, glucose was the preferred sugar for 2-PE biosynthesis.

To sum up, our study underscores the potential of microbial biorefineries, particularly those employing *P.* *putida*, as an environmentally sustainable alternative for producing valuable aromatic compounds like 2-PE. The implementation of strain engineering and the utilization of agricultural waste substrates are pivotal strategies identified for enhancing production efficiency and mitigating environmental impact.

## Materials and methods

### Chemicals

l-Phenylalanine (99%), 2-phenylethanol (99%), phenylacetaldehyde (90%), shikimic acid (99%), sodium phenylpyruvate (powder), chorismic acid barium salt (≥ 80%), *o*-toluic acid (99%), *m*-toluic acid (99%), *p*-fluoro-dl-phenylalanine (FPA) (98%), Viscozyme^R^ l-cellulolytic enzyme mixture and ethylmethanesulfonate (EMS) were purchased from Sigma-Aldrich (Merck). Acetonitrile (HPLC grade), d-( +)-xylose and d-( +)-glucose were provided by VWR Chemicals (France).

### Strains, plasmids and growth conditions

Strains and plasmids used in this study are shown in Table 4. *Pseudomonas* DOT-T1E is a solvent-tolerant strain that was originally isolated from a wastewater treatment plant [8]. *Pseudomonas* *putida* CM12-5 is a DOT-T1E derivative that produced phenylalanine and it was previously described [24]. To make *P.* *putida* CM12-5 able to use xylose as a C-source, a derivative of this strain, bearing and *xylE* genes from *E.* *coli* that transports xylose into the cell and the *xylAB,* genes that encode enzymes to convert xylose into xylose-5 phosphate, which is metabolized through the pentose phosphate pathway, was constructed [45]. Efficient utilization of xylose required the inactivation of the glucose/xylose dehydrogenase (Gcd) to avoid the misrouting of xylose to dead-end xylonate [33]. A derivative of CM12-5 with Δ*gcd* and bearing the *xylAB* and *xylE* genes was also available. *Escherichia* *coli* DH5α was used for the cloning experiments and propagation of plasmids (Table 4).

**Table 4:** Strains and plasmids used in this study

Strains/plasmids	Genotype/relevant features	References
Strains *Escherichia* *coli*		
DH5α	Cloning host: F-λ-*endA1* *glnX44*(AS) *thiE1* *recA1* *relA1* *spoT1* *gyrA96*(Nal^R^) *rfbC1* *deoR* *nupG* Φ80(*lacZ*Δ*M15*) Δ(*argF-lac*)*U169* *hsdR17*(*r*_*K*_^–^*m*_*K*_^+^)	[35]
*Pseudomonas* *putida*		
DOT-T1E	Prototroph, Cm^R^, Rif^R^	[8]
DOT-T1EΔ*gcd*	DOT-T1E mutant generated by insertional inactivation of DOT-T1E_2882, Km^R^	[34]
DOT-T1E (*xylABE)*	Wild type harboring plasmid pSEVA633_*xylABE*, Gm^R^	[34]
DOT-T1EΔ*gcd* (*xylABE)*	DOT-T1E Δ*gcd* mutant harboring plasmid pSEVA633_*xylABE*, Km^R^, Gm^R^	[34]
CM12-5	DOT-T1E mutant that produces L-phenylalanine, Rif^R^	[24]
CM12-5Δ*gcd*	CM12-5 mutant generated by insertional inactivation of DOT-T1E_2882, Km^R^	[34]
CM12-5 (*xylABE)*	CM12-5 harboring plasmid pSEVA633_*xylABE*, Gm^R^	[34]
CM12-5Δ*gcd* *(xylABE)*	CM12-5 Δ*gcd* harboring plasmid pSEVA633_*xylABE*, Gm^R^	[34]
PG1E	CM12-5 derivative that grows in the presence of 10 mg mL^−1^ of FPA, Rif^R^	This study
PG2E	CM12-5 (pPHE1) derivative that grows in the presence of 10 mg mL^−1^ of FPA, Rif^R^ Km^R^	This study
*Plasmids*		
pSEVA238	Expression vector: *oriV*(pBBR1), *xylS*/ Pm, Km^R^	[67]
pPHE1	pSEVA238 derivative carrying *pheA*^*fbr*^ gene from *P.* *putida* DOT-T1E	[24]
pSEVA633	Expression vector: *oriV*(pBBR1), *lacZα*-pUC18, Gm^R^	[67]
pSEVA633_*xylABE*	pSEVA633 with *xylABE* genes expressed from the EM7 promoter, Gm^R^	[34]
pSEVA438	Expression vector: *oriV*(pBBR1), *xylS*/Pm, Sm/Sp^R^	[67]
pPE-1	pSEVA438 derivative carrying PP_0968 from *P.* *putida* KT2440, *kdc* from *Rhodospirillum* *rubrum*, and T1E_5478 from *P.* *putida* DOT-T1E, Sm^R^/Sp^R^	[24]
pSEVA433	Expression vector: *oriV*(pBBR1), *lacZα*-pUC18, Sm^R^/Sp^R^	[67]
pPE-2	pSEVA433 derivative carrying PP_0968 from *P.* *putida* KT2440, *kdc* from *Rhodospirillum* *rubrum*, and T1E_5478 from *P.* *putida* DOT-T1E, Sm^R^/Sp^R^	This study

Cm^R^, resistance to chloramphenicol; Gm^R^, resistance to gentamycin; Km^R^, resistance to kanamicyn; Rif^R^, resistance to rifampicin; Sm^R^, resistance to streptomycin; Sp^R^, resistance to spectinomycin. FPA, *p*-fluoro-DL-phenylalanine

*Escherichia* *coli* was routinely grown at 37 ºC in LB medium, while the *Pseudomonas* *putida* strains were grown at 30 ºC in LB medium or in M9 minimal medium [60] with 5 g L^−1^ glucose as the sole carbon source. When indicated M8 minimal medium was used, this is identical to M9 medium except that ammonium chloride was replaced by 10 mM KNO_3_. For liquid cultures 100 mL conical flasks seeded with 20 mL culture medium were incubated in a Kühner thermostat incubator with agitation (200 rpm, 30 ºC). Growth was monitored by following the turbidity of the cultures at 660 nm (OD_660_) in a UV–VIS spectrophotometer Shimadzu 1900i (Kyoto, Japan).

For resting cells assays, the cells were grown in M9 minimal medium with 5 g L^−1^ glucose as the sole carbon source, until the culture reached OD_660_ = 1.0. Then, the cells were centrifuged and washed with 1xM9 and re-suspended in the appropriate medium at OD_660_ = 10.

Pretreated sugar cane straw (PSCS) and corn stover (PCS) were sourced from the Abengoa Bioenergy Biomass Pilot Plant in York, Nebraska, USA. The final dry matter content was determined to be 39.9% for PSCS and 43.3% for PCS, as reported in the compositional characterization by Rocha-Martín et al. [30]. Following acid- and steam-explosion pretreatments, the soluble lignocellulosic substrates exhibited furfural contents ranging from 0.31% to 1.1%, and 5’-hydroxymethylfurfural contents ranging from 0.23% to 0.28%, as documented in the same study (30). PCS and PSCS were then hydrolyzed using VISCOZYME (Sigma-Aldrich) as described by Godoy et al. [45]. The sugar content of these hydrolysates was 1.7 ± 0.1 g L^−1^ glucose and 2.8 ± 0.1 g L^−1^ xylose for PSC and 1.5 ± 0.1 g L^−1^ glucose and 1.4 ± 0.06 g L^−1^ xylose for PSCS. The strains were pre-cultured overnight in M8 minimal medium with 10 mM KNO_3,_ as the N source, then, the cultures were diluted at 0.1 OD_660nm_ in M8 minimal medium with 10 mM KNO_3_ as N source without glucose and 3% (w/v) acid- and heat- pre-treated corn stover (PCS) or acid- and heat- pre-treated sugar cane straw (PSCS) [30]. Note that nitrate was used in these assays, because the pH of the culture medium remain along the fermentation test above 6.1, at which growth of *P.* *putida* takes place, while when ammonium was the N source, the pH of the culture medium dropped to 4.5–5.3, which compromised the survival of the strain. Growth of the strains in this culture was monitored by determining CFU mL^−1^ in solid M9 medium.

Corn syrup was also used as a source of carbon as indicated in the Results section. The corn syrup (purchased from Biosan, Tarragona, Spain) contained about 410 g L^−1^ glucose, 550 g L^−1^ fructose and 40 g L^−1^ sucrose.

### Isolation of l-phenylalanine overproducer *P. putida* mutants

In *Pseudomonas* *putida,* l-phenylalanine is produced through the shikimate pathway (Fig. 1). Mutants of CM12-5 and CM12-5 (pPHE1) strains that overproduce l-phenylalanine were generated using ethylmethanesulfonate (EMS) and selection of clones able to grow in the presence of > 3 mg mL^−1^ *p*-fluor-dl-phenylalanine (FPA), a toxic analogue of l-phenylalanine [61]. Briefly, cells were grown in M9 minimal medium with glucose until the mid-exponential growth phase was reached. Then, 10 aliquots of 100 μL of liquid culture were spread on M9 minimal medium solid plates with glucose as the C source and 3 mg mL^−1^ FPA, and left to dry for a few minutes. Then, a filter containing 30 mg of EMS was deposited on each plate and incubated at 30ºC. After 48 h, the colonies appearing within the inhibition halo were considered potential mutants and were picked and tested for growth in plates of M9 minimal medium with glucose and increasing concentrations of FPA (from 3 to 10 mg mL^−1^ FPA). Those mutants able to grow at high FPA concentrations > 3 mg mL^−1^ were selected as potential l-phenylalanine overproducers. One clone that tolerated 10 mg mL^−1^ FPA of each strain, and confirmed to produce l-phenylalanine, was chosen for further analysis. These clones were named PG1E and PG2E, the latter bearing the pPHE1 plasmid.

### Induction of the expression of Ehrlich pathway genes in pPE-1

The construction of pPE-1 plasmid bearing the three genes involved in 2-PE synthesis through the Ehrlich pathway, i.e. histidinol phosphate transferase (PP_0967 from *P.* *putida* KT2440), phenylpyruvate decarboxylase (*kdc* from *Rhodospirillum* *rubrum*) and a native alcohol dehydrogenase (T1E_5478, *adh* from *P.* *putida* DOT-T1E) was described by Molina-Santiago et al. [24]. The three genes were cloned as an operon and their expression was driven from the XylS/*o*-toluic inducible Pm promoter [50] with 1 mM *o-*toluate.

### Analytical methods

l-Phenylalanine, 2-phenylethanol (2-PE), and other aromatic intermediates present in culture supernatants were identified and quantified using an elution gradient protocol, with an Agilent 1260 HPLC–DAD system (see Godoy et al. [45]). l-Phenylalanine and 2-PE were both monitored at 215 nm, and their elution times were 5 and 10 min, respectively. Glucose, fructose and xylose consumptions were analyzed enzymatically as described by Godoy et al. [45].

### Bio-informatic analysis

The *Pseudomonas* *putida* PG2E genome was sequenced by Stab Vida Lda (Caparica, Portugal and SECUGEN (Madrid)). Single-nucleotide polymorphisms (SNPs) were identified by comparing sequencing reads from the PG2E strain against the *P.* *putida* DOT-T1E reference genome (GCA_026183455.1) with Snippy v4.6.0 [62] that uses BWA MEM (0.7.17) [63] and Freebayes (v1.3.6) [64] with parameters, mincov = 10, minqual = 100 and mapqual = 60. Structural variants were analyzed using sorted bam files with Samtools v.1.16.1 [65] and inspected using IGV v2.15.2 [66].

### Supplementary Information

**Additional file 1: Figure S1.** Comparative growth of *P.* *putida* DOT-T1E, CM12-5 and PG2E strains in the presence of glucose or phenylacetate as C source. Growth of the three *P.* *putida* strains was tested in M9 minimal medium with 0.5% (w/v) glucose or 10 mM phenylacetate as C-source. Turbidity (right side) in liquid medium was measured 24 h after inoculation, while growth on solid medium was observed after 24 h incubation of streaked cells on solid M9 medium with either glucose or phenylacetate.

**Additional file 2: Table S1.** SNPs found in *P.* *putida* PG2E genome with respect to *P.* *putida* DOT-T1E.

**Additional file 3: Table S2.** Identification of mutated genes related with L-phenylalanine metabolism in *P.* *putida* PG2E as compared to *P.* *putida* DOT-T1E strain.

**Additional file 4: Table S3.** Chemical composition of the soluble fraction of acid- and steam- explosion pretreated corn stover (PCS) and sugar cane straw (PSCS). Data are taken from Rocha-Martin et al. [30].

## Data Availability

The data sets generated or analyzed during this study are included in this published article and its supplementary materials.
