# Supplementary figures and images for: Biosynthesis of fragrance 2-phenylethanol from sugars by Pseudomonas putida
Source: Biotechnol Biofuels Bioprod. 2024 Apr 2;17:51. doi: 10.1186/s13068-024-02498-1 (PMC10986128; doi:10.1186/s13068-024-02498-1)

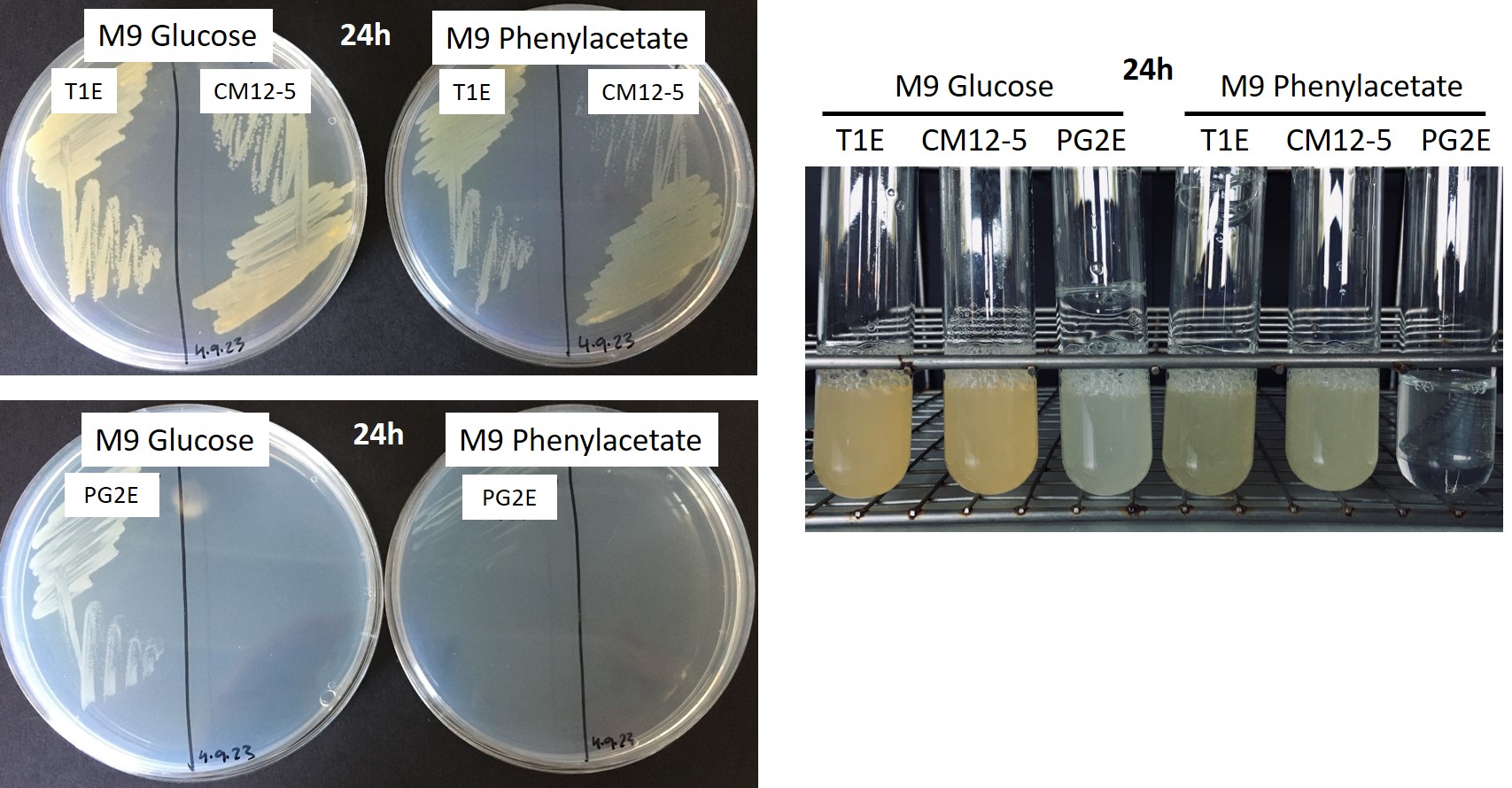

Supplement: Supplementary file 1 — Additional file 1: Figure S1. Comparative growth of P. putida DOT-T1E, CM12-5 and PG2E strains in the presence of glucose or phenylacetate as C source. Growth of the three P. putida strains was tested in M9 minimal medium with 0.5% (w/v) glucose or 10 mM phenylacetate as C-source. Turbidity (right side) in liquid medium was measured 24 h after inoculation, while growth on solid medium was observed after 24 h incubation of streaked cells on solid M9 medium with either glucose or phenylacetate. [file 13068_2024_2498_MOESM1_ESM.jpg]
