# Supplementary material for: Biosynthesis of fragrance 2-phenylethanol from sugars by Pseudomonas putida
Source: Biotechnol Biofuels Bioprod. 2024 Apr 2;17:51. doi: 10.1186/s13068-024-02498-1 (PMC10986128; doi:10.1186/s13068-024-02498-1)
Supplement: Supplementary file 3 — Additional file 3: Table S2. Identification of mutated genes related with L-phenylalanine metabolism in P. putida PG2E as compared to P. putida DOT-T1E strain. [file 13068_2024_2498_MOESM3_ESM.docx]

**Table S2.** Identification of mutant genes related with phenylalanine metabolism in *P. putida* PG2E as compared to *P. putida* DOT-T1E strain.

|  |  | **Actual locus^a^** | **Old locus^b^** | **Gene** | **Function** | **Reference** |
| --- | --- | --- | --- | --- | --- | --- |
| Directed mutagenesis | Phenylalanine metabolism | DOT-T1E_02168 | T1E_0122 | *hppD* | 4-Hydroxyphenylpyruvate dehydrogenase | [24] |
|  |  | DOT-T1E_03002 | T1E_3356 | *hppD* | 4-Hydroxyphenylpyruvate dehydrogenase | [24] |
|  |  | DOT-T1E_00838 | T1E_4057 | *phhA* | Phenylalanine 4-monooxygenase | [24] |
|  |  | DOT-T1E_01652 | T1E_1753 | *katG* | Catalase/peroxydase HPI | [24] |
|  |  | DOT-T1E_02796 | T1E_1616 | *styD_2* | Phenylacetaldehyde dehydrogenase | [24] |
|  |  |  |  |  |  |  |
| Missense variants | Phenylalanine metabolism | DOT-T1E_02335 | T1E_5512 | *xylB_2* | Aryl-alcohol dehydrogenase | This work |
|  |  | DOT-T1E_02359 | T1E_5534 | *paaH_2* | 3-Hydroxyadipyl-CoA dehydrogenase | This work |
|  |  | DOT-T1E_00975 | T1E_3087 | *dadA1_2* | D-Amino acid dehydrogenase 1 | This work |
|  |  | DOT-T1E_02417 | T1E_5594 | *paaK* | Phenylacetate-CoA ligase | This work |
|  |  | DOT-T1E_02418 | T1E_5595 | *paaA* | 1,2-Phenylacetyl-CoA epoxidase, subunit A | This work |
|  |  |  |  |  |  |  |
|  | Phenylalanine, tyrosine and tryptophan biosynthesis | DOT-T1E_02902 | T1E_2295 | *quiA* | Quinate/shikimate dehydrogenase (quinone) | This work |
|  |  | DOT-T1E_03824 | T1E_3018 | *pheA* | Bifunctional chorismate mutase / prephenate dehydratase | This work |

^a^According to CP110782 (NCBI); ^b^According to CP003734 (NCBI)
