# Supplementary material for: Biosynthesis of fragrance 2-phenylethanol from sugars by Pseudomonas putida
Source: Biotechnol Biofuels Bioprod. 2024 Apr 2;17:51. doi: 10.1186/s13068-024-02498-1 (PMC10986128; doi:10.1186/s13068-024-02498-1)
Supplement: Supplementary file 4 — Additional file 4: Table S3. Chemical composition of the soluble fraction of acid- and steam- explosion pretreated corn stover (PCS) and sugar cane straw (PSCS). Data are taken from Rocha-Martin et al. [30]. [file 13068_2024_2498_MOESM4_ESM.docx]

**Table S3.** Chemical composition of the soluble fraction of acid- and steam-explosion pretreated corn stover (PCS) and sugar cane straw (PSCS). Data are taken from Rocha-Martín et al. [30].

| **Component** | **PCS (% DM)** | **PSCS (%DM)** |
| --- | --- | --- |
| Acetic acid | 1.19 ± 0.01 | 2.08 ± 0.07 |
| Furfural | 0.31 ± 0.05 | 1.1 ± 0.08 |
| 5-hydroxymethylfurfural | 0.23 ± 0.07 | 0.28 ± 0.06 |
| Glucose | 1.1 ± 0.1 | 2.96 ± 0.05 |
| Xylose | 10.12 ± 0.16 | 8.06 ± 0.1 |

% DM, percent of total content on a dry matter basis
